# Kidney Function Modulates Gut Microbial Metabolism

**DOI:** 10.3390/toxins18040176

**Published:** 2026-04-04

**Authors:** Mara Lauriola, Sophie Valkenburg, Sander Dejongh, Ward Zadora, Hubert Krukowski, Pieter Evenepoel, Jeroen Raes, Ricard Farré, Griet Glorieux, Björn Meijers

**Affiliations:** 1 Nephrology and Renal Transplantation Research Group, Department of Microbiology, Immunology and Transplantation, KU Leuven, 3000 Leuven, Belgium; 2 Translational Research Center for Gastrointestinal Disorders (TARGID), KU Leuven, 3000 Leuven, Belgium; 3 Department of Nephrology and Renal Transplantation, UZ Leuven, 3000 Leuven, Belgium; 4 Nephrology Section, Department of Internal Medicine and Paediatrics, Ghent University Hospital, 9000 Ghent, Belgiumgriet.glorieux@ugent.be (G.G.); 5 Laboratory of Molecular Bacteriology, Department of Microbiology, Immunology and Transplantation, Rega Institute for Medical Research, KU Leuven, 3000 Leuven, Belgium; 6 Laboratory of Bioinformatics and (Eco-) Systems Biology, VIB, Center for Microbiology, 3000 Leuven, Belgium

**Keywords:** targeted metabolite analyses, colon proteolytic fermentation, tryptophan, gut microbiota, chronic kidney disease, gut–kidney axis

## Abstract

Growing evidence suggests that chronic kidney disease (CKD) profoundly disrupts gut microbiome and its activity. This study explores how CKD affects colon microbial metabolism, focusing on (1) the representativeness of fecal metabolomics, (2) saccharolytic and proteolytic fermentation metabolites, and (3) the gut microbiome’s role in the partitioning of tryptophan in its metabolic pathways. Tryptophan’s main metabolic pathways include the indolic and the kynurenine pathways, which lead, respectively, to the formation of indoxyl sulfate and kynurenine, both contributing to uremic toxicity. Using a rat model of CKD, we evaluated whether fecal concentrations of microbial compounds, on which most studies are based, reflect the colonic concentrations in contact with the gut mucosa. Thus, we quantified the concentration and content of amino acids, indole, p-cresol, and also short-chain fatty acids, in different colon sections. We demonstrated that CKD promotes increased proteolytic fermentation and an augmented tryptophan partitioning into both the indolic and kynurenine pathways. Depletion of the indolic pathway obtained upon antibiotic treatment leads to a further enhancement of the kynurenine pathway.

## 1. Introduction

Chronic kidney disease (CKD) is characterized by structural and functional gastrointestinal alterations and by the accumulation of waste products in the blood [1]. These two elements have been closely studied as part of these accumulating solutes derived from microbial metabolism in the large intestine. Indeed, the colon hosts the highest bacterial concentration (10^7^–10^8^/mL), while the upper intestine supports fewer bacteria (10^3^–10^4^/mL) due to its acidic environment [2]. Some microbial metabolites produced in the gut exert harmful effects. Thus, they are considered uremic toxins (UTs). The so-called gut–kidney axis refers to the complex bidirectional interactions between gut and kidney physiology [3]. CKD is linked to gut microbiota dysbiosis [4], i.e., a disruption in the gut microbial communities [5], which is characterized by a reduced abundance of beneficial butyrate-producing bacteria (i.e., *Roseburia*, *Prevotella*, and *Bacteroides*), and by an increase in pro-inflammatory microbes (*Proteobacteria*, *Actinobacteria*) [4]. Proteobacteria (e.g., *Escherichia coli*, *Salmonella*, *Desulfovibrio*) can damage the gut barrier and produce the UTs precursors indole and p-cresol [4]. Studies reported more bacteria possessing urease, uricase, and indole and p-cresol-forming enzymes, and fewer with butyrate-forming enzymes, in patients with kidney failure (KF) [6], suggesting a shift toward proteolytic microbes.

However, estimating microbial metabolic activity from gut microbiome composition studies is challenging [7]. Apart from gut microbial composition, other factors drive microbial metabolic activity. Several of these, e.g., diet and drug therapy, the use of pre-, pro-, and synbiotics, as well as the use of antibiotics, interfere with gut microbial metabolism [8]. These factors are common in individuals with CKD. Using untargeted metabolomics analysis of stool samples from patients on hemodialysis, their household contacts and unrelated age-matched controls [9], we previously found an influence of the CKD-adapted diet. Animal models provide a controlled setting to study the effect of CKD per se on gut microbial metabolism [9].

So far, studies have primarily relied on the analysis of fecal samples [10]. While those are easily accessible and offer valuable insights into the end products of microbial metabolism, they may provide an incomplete picture of its complexity along the gut. A landmark post mortem human study showed that short-chain fatty acids (SCFAs) are primarily produced in the colon [11], with total concentrations varying by colon segment. This suggests fecal metabolite levels may not reflect the concentrations in contact with the colon mucosa. While fecal SCFAs decline in CKD patients [12,13], there is no data on the effects of kidney dysfunction on their colon levels.

In addition to generating SCFA through saccharolytic fermentation, gut microbes are actively involved in proteolytic fermentation. Tryptophan metabolism is implicated in generating UTs that contribute to cardiovascular complications in CKD [14]. Tryptophan is metabolized through three main pathways: microbes metabolize tryptophan towards indole, while the host processes it via the kynurenine- and the serotonin pathways. Most of the absorbed tryptophan is metabolized by the host into kynurenine by enzymes such as indoleamine 2,3-dioxygenase (IDO) and tryptophan 2,3-dioxygenase (TDO).

In CKD, it is unclear whether feces accurately reflect colonic saccharolytic and proteolytic activity of the gut microbiome. To what extent CKD influences the microbial metabolism in the colon remains to be demonstrated, and how this interacts with endogenous metabolic pathways is unknown. Therefore, in this study we aimed (1) to examine whether fecal metabolomics reflects colon microbial metabolism of several microbial metabolites along the colon, from cecum to rectum, (2) to study whether experimentally induced CKD causes a shift from saccharolytic to proteolytic fermentation, (3) to explore if CKD and gut microbiota depletion by antibiotic administration in CKD affect tryptophan metabolic pathways contributing to uremic toxicity.

## 2. Results

### 2.1. Experiment 1

#### Fresh Fecal Concentrations of Proteolytic Metabolites Do Not Fully Reflect Their Colonic Concentrations

**Figure 1:**
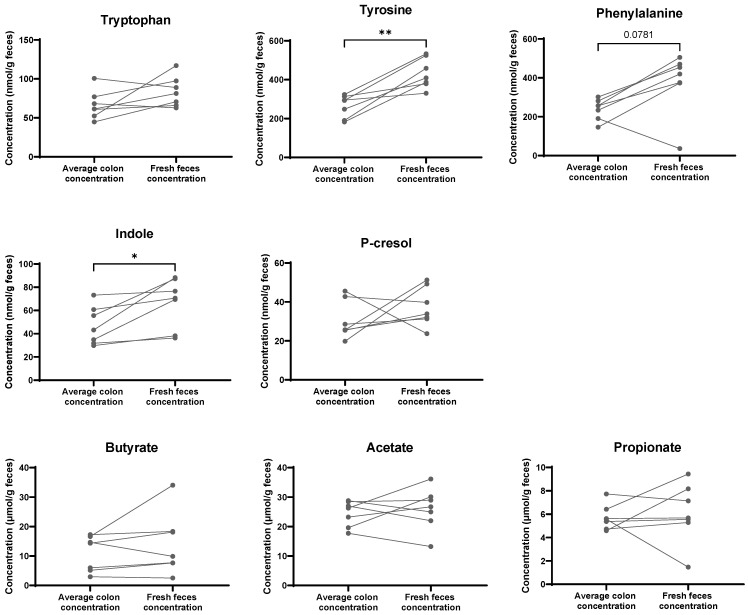
Comparison between rats’ average metabolite concentration along the colon (calculated by dividing the total content of each compound (nmols or µmols) from proximal to rectum by the total fecal pellet weight (mg)) and fresh feces concentration (n = 7 of which n = 3 CKD and n = 4 sham rats). Paired *t*-test (normally distributed data) or Wilcoxon test (skewed data) was used. The * indicates the significant effect of the location (colon content vs. feces) on the concentration. * *p* < 0.05; ** *p* < 0.01.

First, we explored whether fecal metabolite concentrations in fresh feces are representative of colonic concentrations. We collected fresh feces and pellets along the length of the colon. We measured concentrations of saccharolytic fermentation metabolites, i.e., the SCFAs acetate, butyrate, and propionate, as well as proteolytic fermentation metabolites indole and p-cresol. We also measured the precursor AAs tryptophan, tyrosine, and phenylalanine. We calculated the average concentration of the colon content and compared it with the fresh feces’ concentration. P-cresol, tryptophan, and SCFA concentrations did not differ significantly between fresh feces and the colon content when CKD and sham rats were combined. Phenylalanine levels tended to be higher in the feces than in the colon (*p* = 0.07). The fecal concentrations of indole and tyrosine were higher in the feces than in the colon (*p* = 0.02 and *p* = 0.002, respectively) (Figure 1).

Next, we compared concentrations across the different colon sections with the fecal concentrations. The fecal concentration of tryptophan was higher than the rectum concentration (*p* = 0.009), and fecal p-cresol was higher than proximal colon p-cresol (*p* = 0.03) (Supplementary Figure S1).

### 2.2. Experiment 2

#### 2.2.1. Reduced Kidney Function Promotes Proteolytic Fermentation

We performed a 5/6^th^ nephrectomy to induce experimental CKD. Estimated GFR was reduced by, on average, 73% (*p* = 0.0003) (Supplementary Figure S2). We studied proteolytic and saccharolytic fermentation activity in samples collected from the different colon segments. The content was calculated as the product of concentration and pellet weight (Supplementary Figure S3).

**Figure 2:**
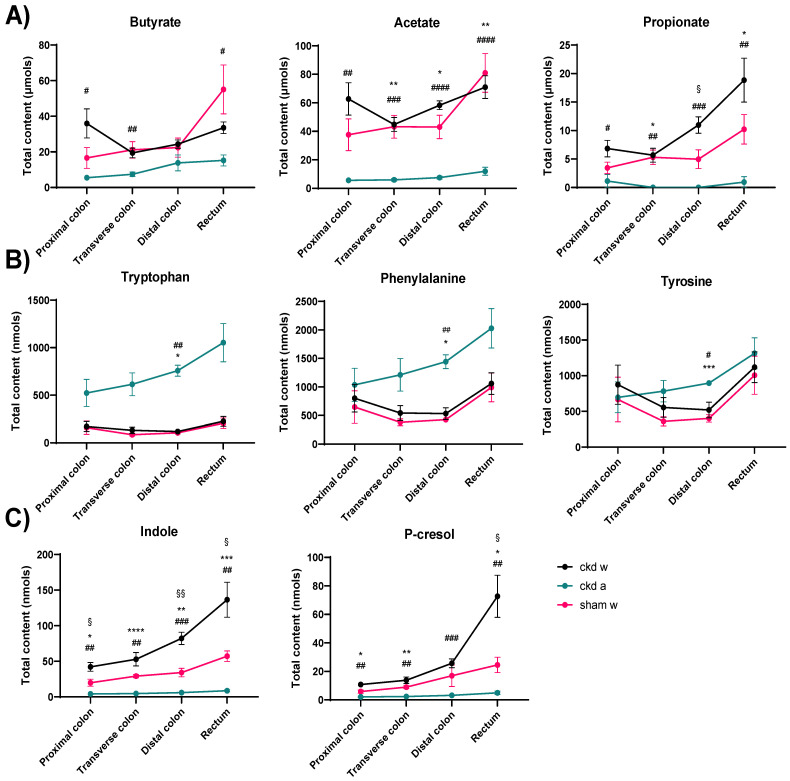
Colon content by section (content calculated based on concentrations along the colon multiplied by the weight of the colon pellet) in (**A**) short-chain fatty acids (SCFAs); (**B**) amino acids; (**C**) uremic toxin (UTs) precursors. CKD W = CKD rats given control water; CKD A = CKD rats given antibiotics; SHAM w = sham rats given control water. * CKD A vs. SHAM W. ## = CKD A vs. CKD W. § = CKD W vs. SHAM W. * or # or § = *p* < 0.05; ** or ## or §§ = *p* < 0.01; *** or ### = *p* < 0.001; **** or #### = *p* < 0.0001. A mixed-effects model with Geisser–Greenhouse correction (matched values) with Tukey’s multiple comparisons test was applied. Mean ± SEM are shown.

**Figure 3:**
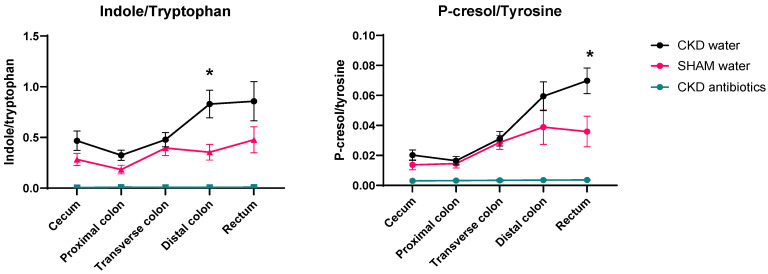
Indole/tryptophan and p-cresol/tyrosine ratio in colon pellet content in rats. Two-way repeated-measures ANOVA was used to compare untreated CKD and SHAM rats. Mann–Whitney or Unpaired Student *t*-test was used to compare these 2 groups for each section. Mean ± SEM are shown (normally distributed data). * CKD vs. SHAM. * *p* < 0.01.

We measured the amino acid precursors tyrosine, tryptophan, and phenylalanine, as well as their fermentation products indole and p-cresol. Indole and p-cresol generation (as estimated by both concentration and content) were higher in the large intestine of CKD rats than in the colon of sham rats (Figure 2 and Supplementary Figures S4 and S5). In contrast, the content and concentrations of their precursor AAs, i.e., tryptophan, tyrosine, and phenylalanine, were similar between CKD and controls (Figure 2 and Supplementary Figures S4 and S5). To further examine whether proteolytic fermentation activity is indeed altered and not a mere reflection of an increased influx of the amino acid precursors, we calculated the concentration ratios of indole over tryptophan and of p-cresol over tyrosine. Both ratios are increased in CKD animals compared to control animals (*p* = 0.03 for indole/tryptophan and *p* = 0.047 for p-cresol/tyrosine), with higher levels in the distal colon and rectum, respectively (Figure 3).

We also examined SCFA concentrations and content. Concentrations tended to be reduced from the proximal to the distal colon (Supplementary Figure S5A), while this tendency was lost when considering the total content (Figure 2A). When comparing CKD with sham rats, concentrations and content of SCFAs did not differ (Supplementary Figure S5), except for propionate content, which was higher in the distal colon of CKD rats (*p* = 0.04) (Figure 2A).

In the group of CKD rats (n = 8), eGFR was positively correlated with butyrate concentrations in the proximal colon and rectum (*p* = 0.046, Spearman r = 0.74; and *p* = 0.02, Spearman r = 0.86). Interestingly, among the CKD and sham rats, we found a strong correlation between colon p-cresol and propionate content (*p* = 0.002; Spearman r = 0.93), suggesting they may share a common microbial origin or that the microbes that produce them are interdependent.

Taken together, we show here that CKD promotes the indolic pathway of tryptophan metabolism and, more generally, leads to enhanced proteolytic fermentation.

#### 2.2.2. Reduced Kidney Function Increases Tryptophan Metabolism Through Both the Indolic and Kynurenine Pathways

**Figure 4:**
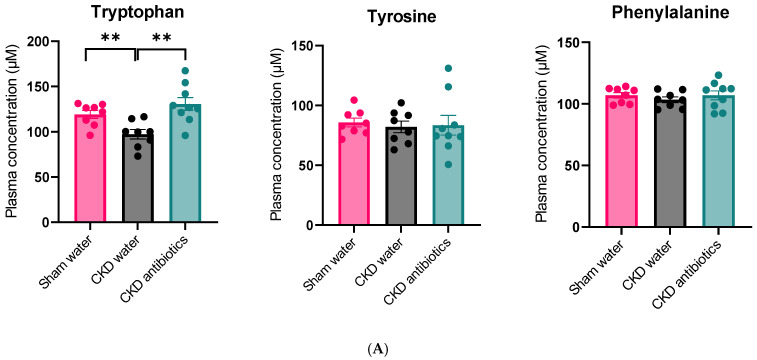

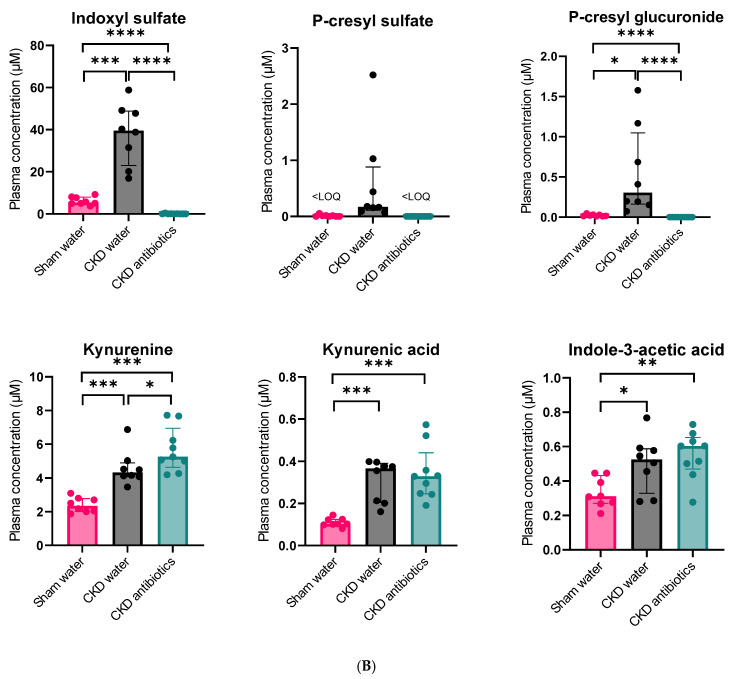
Plasma metabolite levels. (**A**) Plasma amino acid concentrations in control animals, CKD animals, and CKD animals treated with antibiotics. (**B**) Plasma amino acid-derived metabolites in rats. Mann–Whitney or Unpaired Student *t*-test was used for two-by-two comparisons. Median ± interquartile range is shown for skewed data. Mean ± SEM are shown for normally distributed data. * *p* < 0.05; ** *p* < 0.01, *** *p* < 0.001; **** *p* < 0.0001.

**Figure 5:**
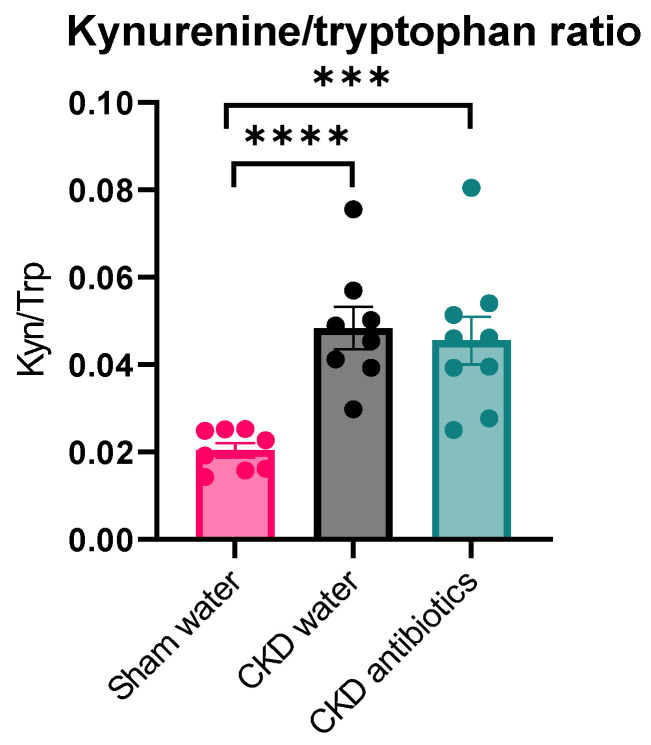
Plasma kynurenine/tryptophan ratio among the three groups. An unpaired Student *t*-test was used for two-by-two comparisons. Mean ± SEM is shown. CKD W = CKD rats given control water; CKD A = CKD rats given antibiotics; SHAM w = sham rats given control water. *** *p* < 0.001; **** *p* < 0.0001.

Next, we studied the precursor AAs as well as their proteolytic fermentation co-metabolites in the blood and urine of these animals. We observed lower plasma tryptophan concentrations in CKD animals, whereas plasma concentrations of both tyrosine and phenylalanine were similar between CKD and controls (Figure 4). As expected, experimental loss of kidney function resulted in increased plasma concentrations of IS, PCS, PCG, kynurenine, KYNA, and I3AA. The conversion of tryptophan to kynurenine is rate-limited by the first enzymatic conversion by tryptophan 2,3-dioxygenase (TDO) in the liver and indoleamine 2,3-dioxygenase 1 (IDO1), mainly in immune cells. The kynurenine/tryptophan ratio is used to estimate the combined effect of the TDO and IDO enzymatic activities. This ratio was higher in CKD animals than in controls (*p* < 0.0001) (Figure 5).

Metabolic caging was used to collect 24 h urine. We did not find any difference between CKD not treated with antibiotics and sham rats in 24 h-urinary excretion of the different metabolites, except for kynurenine (*p* = 0.001), which was increased in the CKD group (Supplementary Figure S6). These findings suggest an increased conversion of tryptophan into kynurenine in CKD.

When considering all the rats combined (untreated CKD and sham), 24 h-urinary PCG and PCS strongly correlated with total colon content of p-cresol (*p* = 0.02, Spearman r = 0.61 and *p* = 0.03, Spearman r = 0.65, respectively), with total content of tyrosine (*p* = 0.008, Spearman r = 0.66 and *p* = 0.026, Spearman r = 0.58) and with p-cresol concentration in the rectum (*p* = 0.03, Spearman r = 0.60 and *p* = 0.02, Spearman r = 0.64). In CKD rats not treated with antibiotics, the correlation with total p-cresol colon content was even stronger (*p* = 0.01, Spearman r = 0.86 for both). Among CKD rats, 24 h-urinary IS correlated with indole concentration in the transverse colon (*p* = 0.037; Spearman r = 0.76).

### 2.3. Suppression of Colonic Microbial Metabolism Increases Plasma Tryptophan and Its Metabolism via the Kynurenine Pathway

The above findings indicate complex effects of loss of kidney function on tryptophan metabolism, with both an increased microbial conversion into indole and an increased systemic conversion into kynurenine. As the interplay between microbial metabolism and kidney function is complex, we aimed to assess the isolated effects of kidney function on systemic tryptophan metabolism. We used broad-spectrum antibiotics to suppress the microbial metabolism of tryptophan.

Antibiotics resulted in a 16-fold reduction in microbial counts. In colonic pellets, antibiotic treatment reduced the content of all three studied SCFAs. Also, this resulted in higher distal colon pellet content of the studied amino acids, i.e., tryptophan, tyrosine, and phenylalanine, whereas the content of the microbial metabolites indole and p-cresol was lower (Figure 2).

We also studied these compounds in plasma and 24 h urine. Plasma tryptophan concentrations increased, while IS and PCS concentrations decreased. These findings were mirrored in their 24 h urine excretion (Supplementary Figure S6).

Intriguingly, despite the increased plasma levels of tryptophan, the antibiotic treatment further increased kynurenine concentration in CKD (Figure 4, *p* = 0.04) (differently from sham rats, see Supplementary Data S4). The ratio of kynurenine/tryptophan (indicating TDO/IDO activity) was not affected by antibiotic treatment in CKD rats, suggesting that the observed increase in plasma kynurenine secondary to suppression of the gut microbiome is not driven by increased enzymatic activity but rather by an increased concentration of tryptophan (Figure 5). At the same time, we did not observe differences in kynurenic acid (*p* > 0.99) or I3AA (*p* = 0.49) compared with the untreated CKD group. However, the latter may derive from the chow itself, as we specified in our previous review [15].

## 3. Discussion

**Figure 6:**
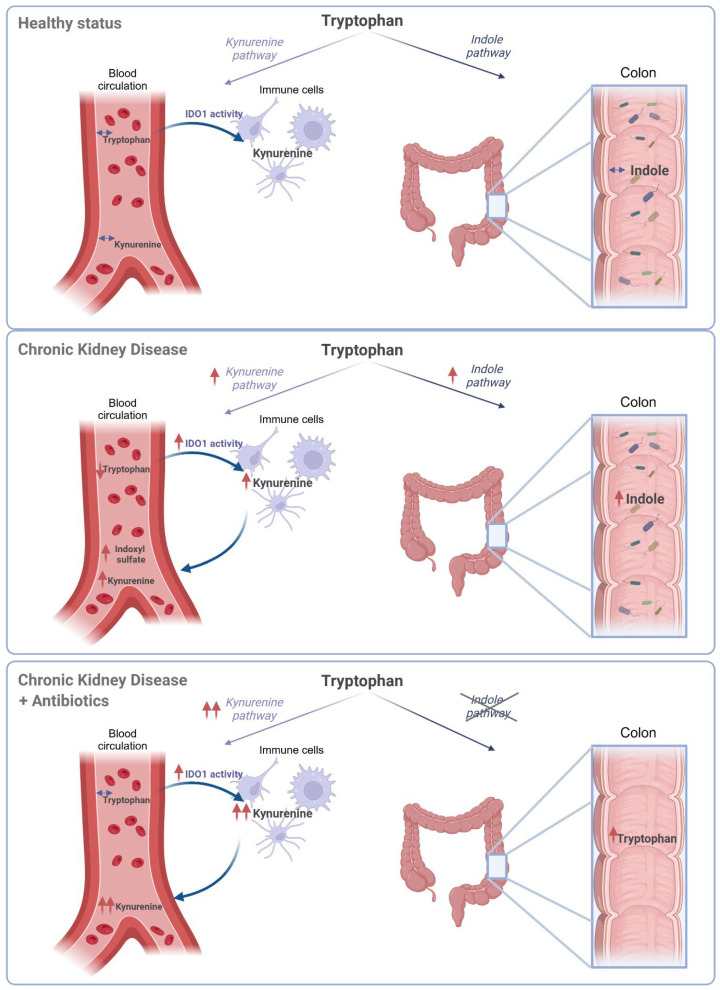
Overview of the findings about tryptophan metabolism. In a healthy condition, tryptophan follows the indole, kynurenine, or serotonin pathways (the last one not represented). In CKD, plasma levels of tryptophan are reduced, while its metabolization into both the indolic and kynurenine pathways is enhanced. Upon antibiotics administration, suppression of the gut microbiome restores plasma tryptophan levels in CKD. While the indolic pathway is drastically reduced, an increase in tryptophan metabolism via the kynurenine pathway was observed.

Here, we demonstrate that the concentrations of various fermentation metabolites in fecal matter do not consistently reflect their actual levels in the colon. Fecal indole concentrations differ from the actual concentrations along the length of the large intestine, i.e., the concentration in proximity to the intestinal barrier. Second, experimental loss of kidney function promotes proteolytic fermentation, without significant changes in the saccharolytic fermentation. In addition, the effects of kidney function on tryptophan partitioning into its main metabolic pathways are complex. While increased proteolytic fermentation enhances tryptophan metabolism via the indolic pathway, the systemic kynurenine pathway is also upregulated in CKD. Lastly, we proved that the antibiotic-induced depletion of the gut microbiota in CKD shifts tryptophan metabolism from the indole to the kynurenine pathway, consistent with the microbiota being a major contributor to indole-derived uremic toxin production. However, as kynurenine itself is also a uremic toxin, this shift may still contribute to the vicious cycle driving uremic toxicity (Figure 6).

Microbial metabolism in the large intestine is often probed indirectly by analyzing fecal matter. While fecal concentrations provide valuable insight as the end product of intestinal processes, our findings suggest that they do not necessarily reflect the concentrations of amino acids and their fermentation products throughout the colon. On the one hand, as water is reabsorbed mainly in the first half of the colon, an increased fecal amino acid concentration compared to the colon lumen concentration may be interpreted as their reduced absorption (or reduced gut microbiota utilization) relative to water. On the other hand, the constant levels of SCFAs along the colon and in the feces may indicate stable production and utilization of undigested carbohydrates. We indicate that indole and p-cresol are largely generated in the distal colon and rectum, as suggested by their increase and the decrease in amino acids. In antibiotics-treated animals, we observed a constant increase in amino acid concentrations, presumably due to water reabsorption. The localized variations in proteolytic activity, with an increase in the distal large intestine, extend the observations in deceased non-CKD individuals by Macfarlane et al. [16].

Here, we demonstrate that kidney function modulates microbial proteolytic activity. The colon content of both indole and p-cresol is higher in the group with experimental loss of kidney function, both when analyzed as concentrations and as calculated content (total colon content in Supplementary Figure S4). In addition, we observed higher indole/tryptophan and p-cresol/tyrosine ratios in the CKD animals. These increased ratios likely reflect enhanced microbial tryptophanase and tyrosine decarboxylase activity, or more broadly, increased bacterial proteolytic activity. An alternative explanation for these findings is a reduced absorption of indole and p-cresol in CKD, and/or increased intestinal secretion of IS and PCS, followed by bacterial deconjugation. However, we consider the latter less plausible, given the observed direct correlations between transverse colon concentration of indole and 24 h-urinary excretion of IS, and between colon content of p-cresol and 24 h-urinary excretion of PCS.

Several factors could contribute to increased proteolytic activity. One might speculate that gut dysbiosis, known to occur in CKD, might lead to a higher abundance of indole- and p-cresol producing bacteria. To test this hypothesis, in a previous study [17], anaerobic cultures of fecal samples collected from CKD patients and non-CKD controls showed no difference in the ex vivo capacity to generate p-cresol, indole, and I3AA, indicating that the metabolic potential of the microbiota in patients with CKD is comparable to that of non-CKD fecal donors. Alternatively, this could be attributed to increased substrate availability. However, both the indole/tryptophan and p-cresol/tyrosine ratios are higher in the CKD animals, indicating regulation beyond simple substrate availability.

Our findings underscore the enhanced fermentation of amino acids within the colon in CKD, leading to an elevated production of harmful metabolites, precursors to UTs such as IS and PCS. To the best of our knowledge, this is the first time that an accurate metabolite analysis along the entire colon in an experimental model of CKD has confirmed this.

Conversely, we could not demonstrate an apparent decrease in SCFAs along the colon, except for a tendency towards a reduced butyrate concentration. Moreover, we found a positive correlation between proximal and transverse colon concentrations of butyrate and eGFR. It could be that CKD in our rat models was not severe enough to show any statistical difference. Indeed, we noted from previous experiments that the 5/6^th^ nephrectomy model results in a milder loss of kidney function than the adenine supplementation model, resulting in higher plasma concentrations of creatinine and several UTs. We deliberately opted for the 5/6^th^ nephrectomy model to avoid bias from the adenine supplementation in the diet, which may interfere with the gut microbiota fermentation activity [18]. In addition, the correlation between eGFR and butyrate aligns with the findings from a human study by Steenbeke et al., who reported a similar correlation between kidney function and butyrate fecal concentration in patients with CKD [19].

Lastly, we show here the complex effects of CKD on tryptophan partitioning. Experimental loss of kidney function enhances colonic tryptophan conversion by gut bacteria into indole, possibly leading to further IS accumulation. At the same time, CKD promotes the systemic conversion of tryptophan into kynurenine. Kynurenine exerts inflammatory effects in chronic diseases [20]. Its role in cardiovascular diseases has also been demonstrated by its contribution to vascular inflammation and endothelial dysfunction, both key drivers of atherosclerosis progression. Additionally, elevated kynurenine levels have been linked to blood pressure regulation and the development of arterial stiffness [21,22].

To better understand if and to what extent the activities of the two metabolic pathways are interconnected, we used antibiotics to suppress the gut microbial indolic pathway. Interestingly, I3AA in plasma was not reduced upon antibiotic use. This may be due to the contribution of the grain-based chow itself, as I3AA can originate from bacteria present in plants, as we previously discussed [15]. Instead, tryptophan plasma levels, typically reduced in CKD (as shown here but also by previous authors [23,24]), were restored by suppressing microbial metabolism. We analyzed the combined enzymatic activity of TDO, mainly located in the liver [25], and IDO1, present mostly in circulating myeloid cells [26], by calculating the kynurenine/tryptophan ratio, and we found an increase in CKD rat models, which was unaltered by the antibiotic administration. IDO1 is the main responsible for extrahepatic kynurenine production, and it is upregulated by pro-inflammatory cytokines [27]. This occurs in many diseases, causing inflammation [28]. To rule out local gut IDO activity, we analyzed the relative IDO1 mRNA expression in colon tissue and found no expression. The increased kynurenine plasma concentration is consequent to higher tryptophan availability. Of note, in an additional experiment described in Supplementary Data S4, we observed a lower kynurenine/tryptophan ratio in healthy animals provided with antibiotics. This led to stable plasma kynurenine levels in sham rats. Consequently, the increase in plasma kynurenine concentration in CKD is not solely due to reduced kidney clearance but also to this heightened formation. Thus, we suggest that either due to higher tryptophan absorption when the gut microbiome is depleted or due to higher availability of plasma proteins to bind and retain tryptophan consequent to the lower competition with UTs, e.g., IS or PCS, more tryptophan enters the kynurenine pathway, leading to additional kynurenine in the plasma of CKD rats provided with antibiotics compared to the untreated ones. In other words, in CKD, the gut microbial metabolism reduces either tryptophan absorption or the capability of the body to retain tryptophan in the plasma due to the binding competition with UTs, thereby reducing its conversion to kynurenine.

One potential explanation for increased absorption is that the pH of the intestinal lumen drives these changes. CKD is characterized by an increased intestinal pH due to the increase in urease-producing bacteria, which break down urea into ammonia in the gut [29,30,31]. This alkaline environment may limit the absorptive activity of tryptophan transporters [25]. Thus, depleting ammonia formation due to microbial activity may enhance absorption. On the other hand, Mingrone et al. showed that serum UTs displace the binding of L-tryptophan to human albumin, corroborating the second hypothesis [32].

In summary, we found that fecal amino acids and metabolites do not consistently reflect colonic fermentation, potentially due to variable microbial fermentation rate, metabolite absorption, water reabsorption, and utilization along the colon. As the conclusions of this experiment are limited by the low number of rats, further studies including a larger sample size are needed to confirm these results.

Colonic proteolytic fermentation is increased by experimentally induced CKD, suggesting a role of gut dysbiosis in the plasma accumulation of detrimental uremic toxins. Nonetheless, a limitation of the present study is that microbiota composition was not directly characterized. Therefore, the observed metabolite alterations cannot distinguish between changes in microbial community structure. However, the measured metabolites represent established microbiota-derived products and thus provide a functional readout of microbiota-associated metabolism.

Another limitation is that metabolite concentrations were measured in fresh fecal samples and not in dry matter. Because water reabsorption occurs progressively along the colon, variations in water content may influence metabolite concentrations. However, analysis of fresh fecal material reflects the luminal content to which the intestinal mucosa is exposed and thus provides physiologically relevant information on metabolite levels within the colonic environment.

In general, the gut microbiome plays a dual role in CKD. While its presence enhances the tryptophan indolic pathway, it simultaneously reduces the availability of tryptophan for conversion into kynurenine, another metabolite considered toxic in CKD. Nonetheless, the gut microbiome also increases indoxyl sulfate generation, contributing to a toxic vicious cycle.

Lastly, these results provide relevant insight into gut–kidney crosstalk. Despite the 5/6^th^ nephrectomy model mirroring moderate-advanced CKD, extrapolation to advanced human disease needs further investigation. Nonetheless, these findings highlight the importance of developing therapies targeting gut dysbiosis and bacterial metabolic pathways to restore tryptophan balance and reduce harmful proteolytic fermentation in CKD. While a dietary approach with reduced tryptophan intake may limit the formation of its downstream metabolites, another promising therapy could be the use of engineered bacteria that convert tryptophan into non-toxic or not-absorbable metabolites [33].

## 4. Materials and Methods

### 4.1. Animal Studies

We used male Sprague Dawley rats (Janvier, France) aged 7–8 weeks. CKD was induced through a 5/6^th^ nephrectomy, performed over two surgeries, while the control groups underwent a sham operation. All the rats were euthanized by decapitation 8 weeks after the second surgery. All procedures, described more in details in Supplementary Data S1, were conducted in accordance with Directive 2010/63/EU and were approved by the university’s animal ethics committee (P037/2019).

### 4.2. Experiment 1

To assess whether fresh feces concentrations of amino acids (AAs) and their microbial metabolites reflect their concentrations along the colon, we induced CKD by 5/6^th^ nephrectomy in 3 rats and 4 sham-operated rats.

We collected blood at euthanasia, centrifuged it, and stored the plasma at −80 °C. A sample of fresh feces was collected just before euthanasia. During post mortem, we subdivided the colon into four parts in a standardized manner, and we collected the content of each section separately, in Eppendorf tubes. Specifically, we made an incision at the cecal end and another one 15 cm towards the rectum. This 15 cm segment was then divided into three equally long parts for simplicity, referred to as the proximal, transverse, and distal colon. The remaining portion of the colon was designated as the rectum.

### 4.3. Experiment 2

To assess differences between CKD rats provided or not with antibiotics and control rats, rats were randomly divided into three groups (n = 8 per group). One week post-surgery, one of the CKD groups started receiving drinking water containing a combination of antibiotics (ampicillin 1 g/L, metronidazole 1 g/L, vancomycin 500 mg/L, ciprofloxacin 200 mg/L) for the following 7 weeks, with the antibiotic solution being refreshed every 2–3 days. The other CKD group and the sham group were given fresh water without antibiotics. Seven weeks post-surgery, rats (n = 24) were placed in metabolic cages to collect 24 h urine samples. At euthanasia, blood was collected and processed as for the previous batch of rats. Of note, Experiment 2 originated from a second study, independent from Experiment 1. Cecum, colon, and rectum fecal contents were also collected. Specifically, a spot sample was collected from the cecum, while the colon content of each section was collected as for the other rats. All the intestinal content and fecal samples were weighed, snap-frozen, and stored at −80 °C until analysis.

### 4.4. Measurements

Samples summarized in Supplementary Table S2 were batch-analyzed at the end of the experiment. Plasma creatinine and urea concentrations were determined by enzymatic assay. Estimated GFR (eGFR) was calculated using the recently developed formula validated for rats [34]. Plasma and urine concentrations of the AAs precursors of indole and p-cresol, i.e., tryptophan, phenylalanine and tyrosine, and the amino acid-derived UTs, namely indoxyl sulfate (IS), p-cresyl sulfate (PCS), p-cresyl glucuronide (PCG), kynurenine, kynurenic acid (KYNA) and indole-3-acetic acid (I3AA), were quantified using ultra-performance liquid chromatography-tandem mass spectrometry (UPLC-MS/MS) with a validated methodology, as previously described [35]. The limit of detection (LOD) and the limit of quantification (LOQ) are reported in Supplementary Table S1.

Tryptophan (Trp), tyrosine (Tyr), phenylalanine (Phe), indole, and p-cresol in colon pellets and fresh feces were quantified by HPLC, as previously described and as reported in Supplementary Data S2 and S3, without a filtration step [17]. All chemicals were purchased from Sigma-Aldrich, Germany. In summary, 5 mL of phosphate buffer was added to 1 g of fecal sample, which was vortexed and centrifuged. The supernatant was stored at −80 °C. HPLC separation was achieved using a mobile phase of 50 mM ammonium formate buffer (pH 3.0) and methanol. The concentration of SCFAs in colon pellets and fresh feces was measured as previously reported [19]. The samples were extracted with diethylether, shaken, and centrifuged, and the supernatant was mixed with NaOH before a second extraction. The aqueous phase was extracted, mixed with HCl, and 10 μL were injected into the UPLC system. The mobile phase consisted of phosphoric acid, methanol, and acetonitrile. The detection was set at 210 nm. The concentration of AAs and metabolites was standardized per weight of feces (g), as previously described by Cummings [11,16]. The total content was also calculated by multiplying the concentration by the weight of each section’s content. The ratios indole/tryptophan and p-cresol/tyrosine were calculated as an estimation of the bacterial tryptophanase [36] and tyrosine decarboxylase [37] activities, respectively. Values below LOD or LOQ were substituted with LOD/√2 and LOQ/√2, respectively, for the statistical analysis.

### 4.5. Statistical Analysis

The results were analyzed using GraphPad Prism 10.1. Data distribution (normal or skewed) was sought via the Shapiro–Wilk test. The comparisons between fresh feces and average colon concentration of microbial metabolites were analyzed with a paired *t*-test (normally distributed data) or a Wilcoxon test (skewed data). Fresh feces concentrations between CKD and sham-operated groups were not performed due to low sample size. Plasma and urine differences in uremic toxin levels were sought via unpaired *t*-test, Welch’s test (if the variance was statistically different), or Mann–Whitney test if data was skewed. A mixed-effects model with Geisser–Greenhouse correction (matched values) with Tukey’s multiple comparisons test was applied to compare CKD rats, CKD rats provided with antibiotics and sham rats and the different sections of the colon. Correlation tests between concentrations of plasma and urine uremic toxins and colon microbial metabolites were performed using the Spearman correlation test. Differences were considered significant if the *p*-value < 0.05.

## Data Availability

The data that supports the findings of this study will be made available upon reasonable request to the corresponding author, or it is retrievable from the Supplementary Materials.

## Supplementary Materials

The following supporting information can be downloaded at: https://www.mdpi.com/article/10.3390/toxins18040176/s1, Data S1: Detailed animal study description; Data S2: HPLC methodology used to measure tryptophan, phenylalanine and tyrosine, indole and p-cresol in the fecal content of rats; Data S3: UPLC methodology used to measure short-chain fatty acids in the fecal content of rats; Data S4: Supplementary rats to assess plasma metabolites (plasma concentration of tryptophan and its metabolites). Figure S1:Microbial metabolites and amino acids concentration along the colon and in fresh feces; Figure S2: eGFR among rat groups; Figure S3: Mass of the pellet content along the different sections of the colon in CKD vs sham rats. Figure S4: Total colon content (sum calculated based on concentrations along the colon multiplied by the weight of the colon pellet) in a) amino acids; b) uremic toxins (UTs) precursors; c) short-chain fatty acids (SCFAs); Figure S5; Colon concentration of a) amino acids; b) uremic toxins (UTs) precursors; c) short-chain fatty acids (SCFAs) from cecum to rectum of rats. Figure S6: 24-hour urinary amino acids and derived metabolites in control animals, CKD animals, and CKD animals treated with antibiotics.; Table S1: LOD and LOQ of amino acids and uremic toxins measured with LC-MS/MS in plasma and urine; Table S2: Summary of samples/specimen analyzed.
