# Supplementary material for: Kidney Function Modulates Gut Microbial Metabolism
Source: Toxins (Basel). 2026 Apr 4;18(4):176. doi: 10.3390/toxins18040176 (PMC13119667; doi:10.3390/toxins18040176)
Supplement: Supplementary file 1 [file toxins-18-00176-s001.zip › toxins-4184641-supplementary.pdf]

Supplementary Materials: Kidney Function Modulates Gut Microbial Metabolism

**Mara Lauriola, Sophie Valkenburg, Sander Dejongh, Ward Zadora, Hubert Krukowski ,
Pieter Evenepoel, Jeroen Raes, Ricard Farré, Griet Glorieux and Björn Meijers**

SUPPLEMENTARY DATA

Table of contents

- **Data**
  - Data S1. Detailed animal study description
  - Data S2. HPLC methodology used to measure tryptophan, phenylalanine and tyrosine, indole and p-cresol in the fecal content of rats.
  - Data S3. UPLC methodology used to measure short-chain fatty acids in the fecal content of rats.
  - Data S4. Supplementary rats to assess plasma metabolites: Plasma concentration of tryptophan and its metabolites
- **Supplementary Figures**
  - Figure S1. Microbial metabolites and amino acids concentration along the colon and in fresh feces (n=7 of which n=3 CKD and n=4 sham rats).
  - Figure S2. eGFR among rat groups.
  - Figure S3. Mass of the pellet content along the different sections of the colon in CKD vs SHAM rats.
  - Figure S4. Total colon content (sum calculated based on concentrations along the colon multiplied by the weight of the colon pellet) in a) amino acids; b) uremic toxins (UTs) precursors; c) short-chain fatty acids (SCFAs).
  - Figure S5. Colon concentration of a) amino acids; b) uremic toxins (UTs) precursors; c) short-chain fatty acids (SCFAs) from cecum to rectum of rats.
  - Figure S6. 24-hour urinary amino acids and derived metabolites in control animals, CKD animals, and CKD animals treated with antibiotics.
- **Supplementary Tables**
  - Table S1. LOD and LOQ of amino acids and uremic toxins measured with LC-MS/MS in plasma and urine.
  - Table S2. Summary of samples/specimen analyzed

Data S1. Detailed animal study description.

Rats (7-8 weeks old) were purchased from Janvier, Le Genest- St Isle, France and kept in pairs in individually ventilated (IVC) cages. The rat chow was purchased from Ssniff-Spezialdiäten GmbH, Germany. Rats allocated to the 5/6th nephrectomy group (n=16) underwent two surgeries after being intraperitoneally injected with ketamine (80mg/kg) and xylazine (10mg/kg) to induce anesthesia, and subcutaneously injected with buprenorphine (0.05mg/kg) as analgesic. The first surgery consisted of the ligation of renal arteries, leading to the ischemia of 2/3rd of the left kidney. Seven days later, a nephrectomy of the right kidney was performed. Control rats (n=8) underwent two sham surgeries. The intervention with the antibiotic cocktail in drinking water started one week after the second surgery (week 1). Rats had free access to *ad libitum* fresh water and feed, and they were housed in the KU Leuven Laboratory Animal Center, under a 12-hour light-dark regimen.

Blood was centrifuged at 1200 g for 15 min at 24 °C.

Data S2. HPLC methodology used to measure indole, p-cresol and amino acids in the fecal content of rats.

Sample preparation. Anaerobic phosphate buffer (1.0g/L sodium thioglycolate, 6.8g/L KH2PO4, and 8.8g/L K2HPO4) (Sigma Aldrich) was added to the fecal sample in a ratio of 5 mL per 1 gram of feces, and vortexed for 10min at maximum speed. The fecal suspension was centrifuged at 10 000g for 30min and the supernatant was transferred to another Eppendorf tube and centrifuged for an additional 10 minutes, after which the supernatants was stored at -80°C.

Prior to chromatography, fecal samples were filtered using an Amicon Ultra 0.5 mL filter (Millipore Merck, Darmstadt, Germany; molecular weight cut-off 30 kDa).

HPLC. Chromatographic HPLC separation was carried out at 26°C using an Ultrasphere 5 ODS Guard column (5µm, 45x4.6mm, Hichrom, Reading, UK) and a reversed-phase XBridge C8 column (3.5µm, 150x4.6mm from Waters). A 50 mM ammonium formate buffer (mobile phase A, pH 3.0) and methanol (mobile phase B) was used as mobile phase. The chromatographic separation was carried out using a linear gradient at a flow rate of 1 mL/min, starting with 100% A during the first 3min, followed by a change into 100% B during the next 36min. After holding the latter composition for three minutes, there was a re-equilibration phase. A Waters 2475 fluorescence detector was used to identify p-cresol (λex: 278nm, λem: 304nm), indole (λex: 275nm, λem: 334nm), tyrosine (λex: 275nm, λem: 302nm), phenylalanine (λex: 257nm, λem: 282nm) and tryptophan (λex: 280nm, λem: 348nm).

Data S3. UPLC methodology used to measure short-chain fatty acids in the fecal content of rats.

Standards, Chemicals, and Reagents. Standards, Chemicals, and Reagents Acetic acid (AA, P/N 5.43808), propionic acid (PA, P/N 94425), butyric acid (BA, P/N 19215), and sodium succinate dibasic hexahydrate (P/N S2378) were purchased from Merck (Merck, Darmstadt, Germany) and stored at room temperature (RT). 5 M AA, PA, and BA stock solutions were prepared in HPLC grade water (Biosolve, Valkenswaard, Netherlands), and they were kept for 3 months at 2–8°C). A stock solution of 0.09 M sodium succinate dibasic hexahydrate, which was used as internal standard (IS), was prepared. The combined working solution (WS, containing AA, PA, and BA) of 100 mM was prepared by diluting the stock solution appropriately with phosphate-buffered saline (PBS) (P/N BE17-516F, Lonza, Verviers, Belgium). The 100 mM working solution was used to prepare the working solutions of 25, 10, 5, and 2.5 mM by diluting the 100 mM working solution appropriately with PBS (Lonza). The working solutions were prepared freshly each time a calibration curve was set up.

The following reagents were required for the liquid-liquid extraction: diethylether (Merck), NaOH (Merck), and hydrochloric acid (HCl, 37%) (VWR, Leuven, Belgium). The following chemicals were required for the ultra high performance liquid chromatography [U(H)PLC] analysis: phosphoric acid (VWR), methanol, and acetonitrile (all from Biosolve) and HPLC grade water.

Sample preparation. Of each standard concentration (0.25, 0.5, 1, 2.5, 5, 10, 15 and 25 mM), 400 μL was pipetted into a 10 mL pyrex extraction tube. PBS was used as blank. A control sample was also analyzed.

For all the samples, 20 μL of the IS stock solution (0.09 M sodium succinate dibasic hexahydrate) was added to each sample. The samples were vortex mixed for 5 s and 40 μL of concentrated HCl was added. Thereafter the samples were vortex mixed again for 15 sec. The samples were extracted by adding 2 mL diethylether with the HandyStep pipet (Brandtech Scientific, Essex, CT, USA), vortex mixed for 5 s, manually mixed 3 times and the pyrex extraction tubes were placed horizontally while shaking at RT for 20 min. Before centrifugation (5 min, 2,851g, 20°C), the samples were manually mixed 3 times. After centrifugation, the supernatant was transferred to a second pyrex extraction tube and 200 μL of a 1 M NaOH solution was added. A second extraction was performed by vortex mixing the samples for 5 s, manually mixed 3 times and placed again horizontally, while shaking warm water bath at RT for 20 min. Before centrifugation (5 min, 2,851g, 20°C), the samples were again manually mixed 3 times. The aqueous phase (200 μL) was transferred to a 1.5 mL Eppendorf vial. Of the latter phase, 150 μL was transferred to an HPLC autosampler vial and 30 μL of concentrated HCl was added. After vortex mixing for 15 s and removing air bubbles, 10 μL of the aliquot was injected.

UPLC. The UPLC system consisted of a G7104A quaternary pump with integrated degasser, a G4226A autosampler, a G1330B thermostat, a G1316C column compartment, and a G4212A diode-array detector (DAD), all from Agilent Technologies (Agilent Technologies, Santa Clara, CA, USA). A temperature of 8°C was set for the autosampler. Chromatographic separation was carried out on an XBridge BEH C18 XP column [150 mm x 4.6 mm internal diameter (i.d.)] with a particle size of 2.5 µm (Waters, Milford, MA, USA). The HPLC column was protected by a guard column of the same type (5 mm x 3.9 mm i.d.). The injection volume was 10 µL using a needle wash of 20% methanol in water for 3 sec. The column was thermostated at 30°C. The mobile phase (MP) consisted of 0.2% phosphoric acid in water (A), methanol (B), and acetonitrile (C). The DAD detector was set at a wavelength of 210 nm using a peak width of 5 Hz. Data processing was performed using Open Lab CDS ChemStation Edition for LC & LC/MS Systems Rev C.01.07 SR2 (Agilent Technologies). The LOQ and LOD were 0.25 mM and 0.08mM for each SCFA, respectively.

Data S4. Supplementary rats to assess plasma metabolites

Rationale and main findings

Further animals were added to the study in order to include also a group of sham rats provided with antibiotics to confirm the findings regarding the plasma concentrations of tryptophan metabolites, including the kynurenine/tryptophan ratio. We showed here that the antibiotics increased plasma tryptophan in both healthy and CKD rats but lowered the kynurenine/tryptophan ratio only among the healthy rats, while this ratio remained higher in both the CKD groups. This confirmed that the excess of plasma tryptophan consequent to the use of antibiotics in healthy animals does not lead to an increased kynurenine formation, differently from rats with CKD.

Method description

A total of 35 rats were added (n=10 SHAM water; n=10 SHAM antibiotics; n=7 CKD water; n=8 CKD antibiotics). The same conditions were used for this new batch of rats, including surgical operations, food, housing, light-darkness cycle. This new study was approved under the same trial number by the Ethics Committee.

Sample collection and analysis

Similarly to the other rats, plasma samples of the additional rats were collected at euthanasia, 8 weeks post-surgery, and stored at -80°C until batch analysis. Plasma samples were analyzed for creatinine, urea, amino acids and uremic toxins similarly to the other rats. eGFR was calculated to confirm the loss of kidney function (significant).

Plasma concentration of tryptophan and its metabolites across the four groups.

The results were analyzed by merging data from both batches. Shapiro-Wilk test was used to assess data distribution. To compare plasma concentrations between groups, an unpaired t test or Mann-Whitney test were used for normally distributed and skewed data, respectively. The Welch's test was used in the case of different variance. All the results are illustrated in the figure below. Median and interquartile ranges are shown.

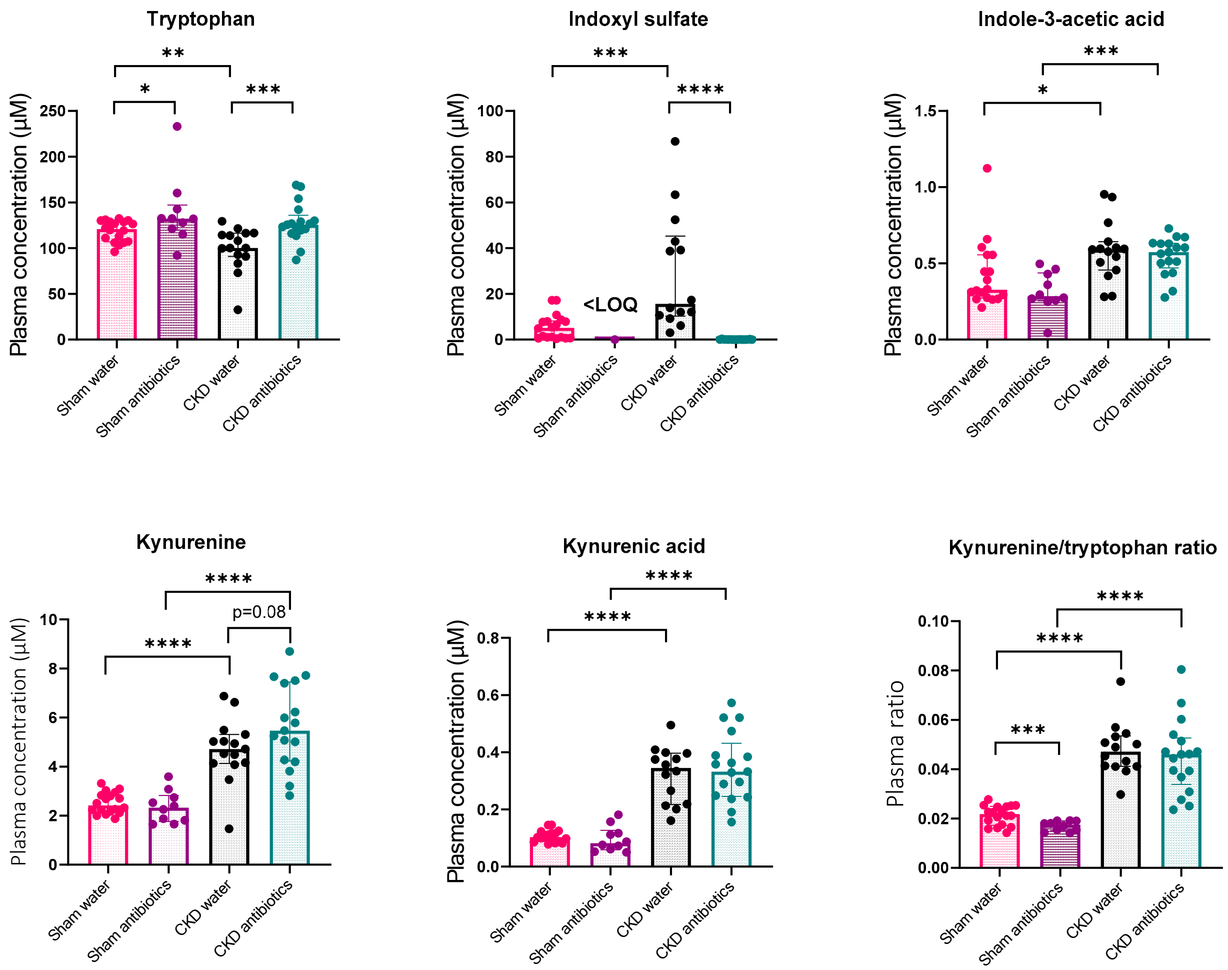

FIGURES

Figure S1

a) Microbial metabolites and amino acids concentration along the colon and in fresh feces (n=7). Normality was sought via Shapiro-Wilk test. Repeated measures ANOVA was used to compare the different sections in the case of normally distributed data, and if $p<0.05$, a Tukey multiple comparisons test was used. Friedman test with Dunn's multiple comparisons test, if $p<0.05$, was used for skewed data. Data is presented as median ± interquartile range. *$p<0.05$; **$p<0.01$

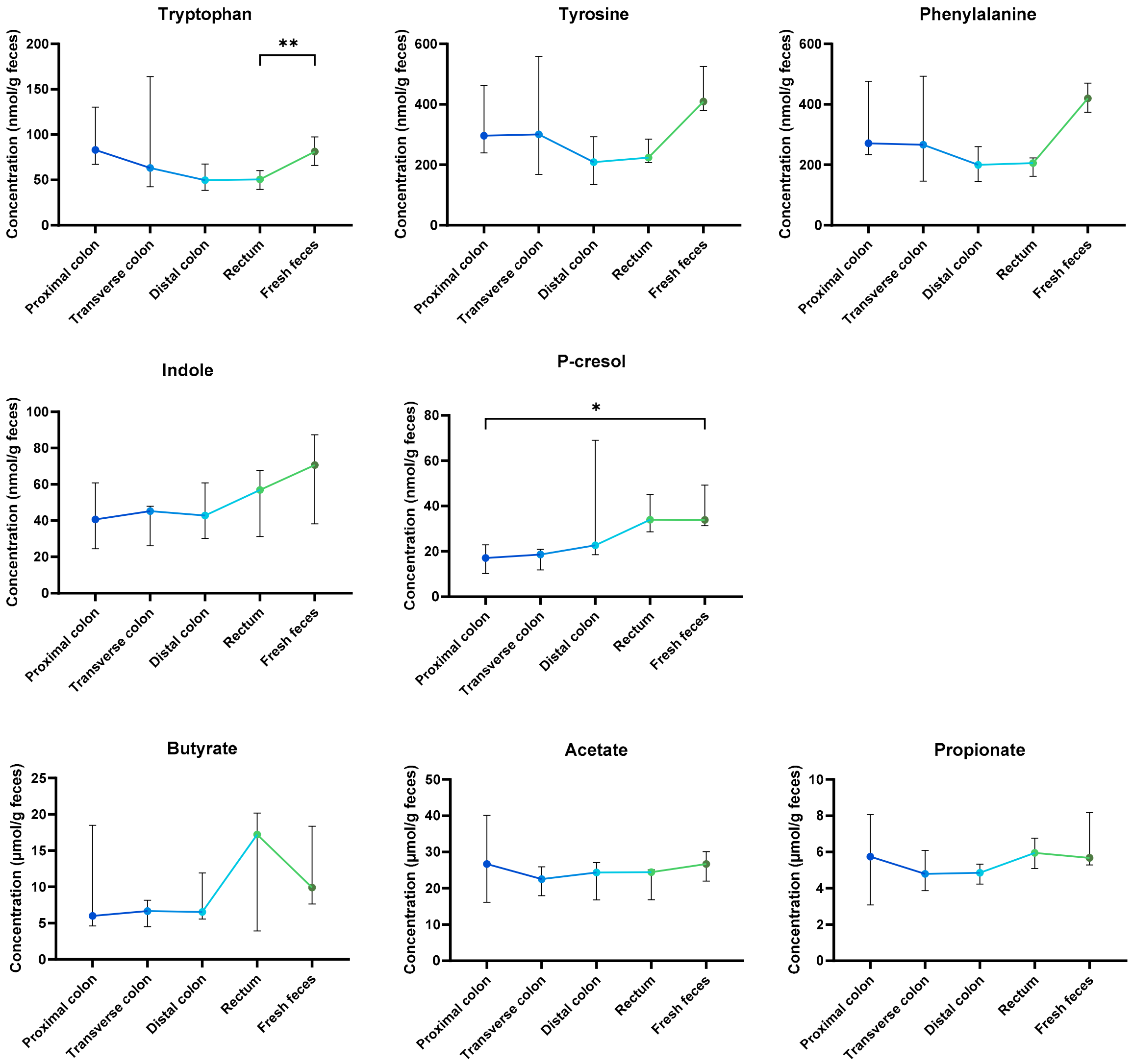

b) Microbial metabolites and amino acids concentration along the colon and in fresh feces divided by CKD (n=3) and SHAM (n=4) rats. Normality was sought via Shapiro-Wilk test. Of note, these graphs include a low number of samples. Despite the low sample size does not allow us to reach conclusions, a repeated measures two-way ANOVA with the Geisser-Greenhouse correction was used (normally distributed data) to compare the different sections between CKD and SHAM rats, and if p<0.05, a Tukey multiple comparisons test was used and shown here with a *. Data is presented as mean ± SEM *p<0.05.

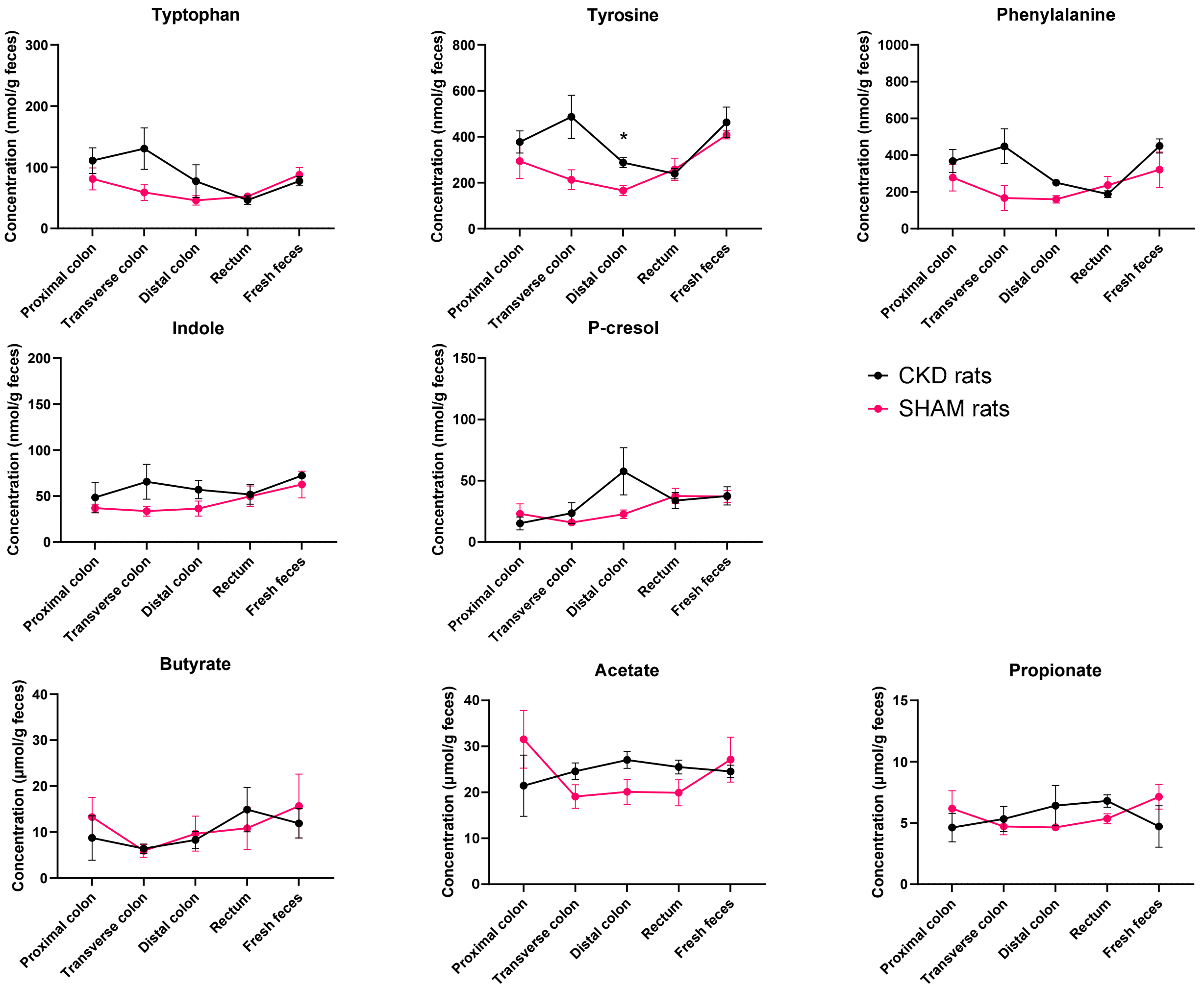

Figure S2. eGFR calculated for the rat groups. Kruskall-Wallis with Dunn's multiple comparisons test was applied. **p<0.01; ***p<0.001;

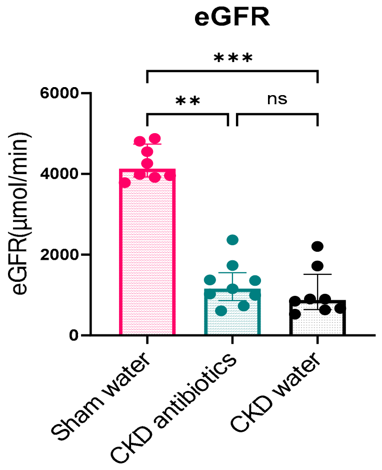

Figure S3. Mass of the pellet content along the different sections of the colon in CKD vs SHAM rats (not treated with antibiotics). Mixed-effects model with the Geisser-Greenhouse correction with Sidak's multiple comparisons test was used. Medians and interquartile ranges are shown. A significant difference was found between the proximal colon content mass of CKD rats compared to SHAM rats (p<0.0001).

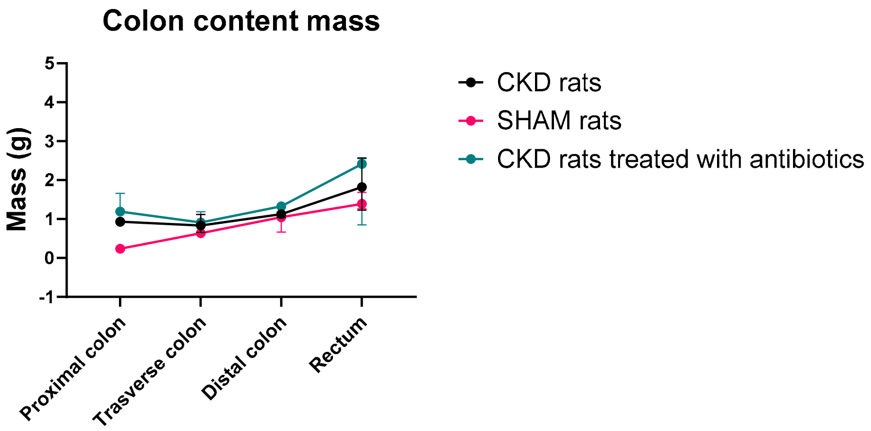

Figure S4. Total colon content (sum calculated based on concentrations along the colon multiplied by the weight of the colon pellet) in a) amino acids; b) uremic toxins (UTs) precursors; c) short-chain fatty acids (SCFAs).*p<0.05; **p<0.01; ***p<0.001; ****p<0.0001. Pairwise comparisons were done by Mann-Whitney or unpaired t test if non-parametric or parametric data distribution, respectively

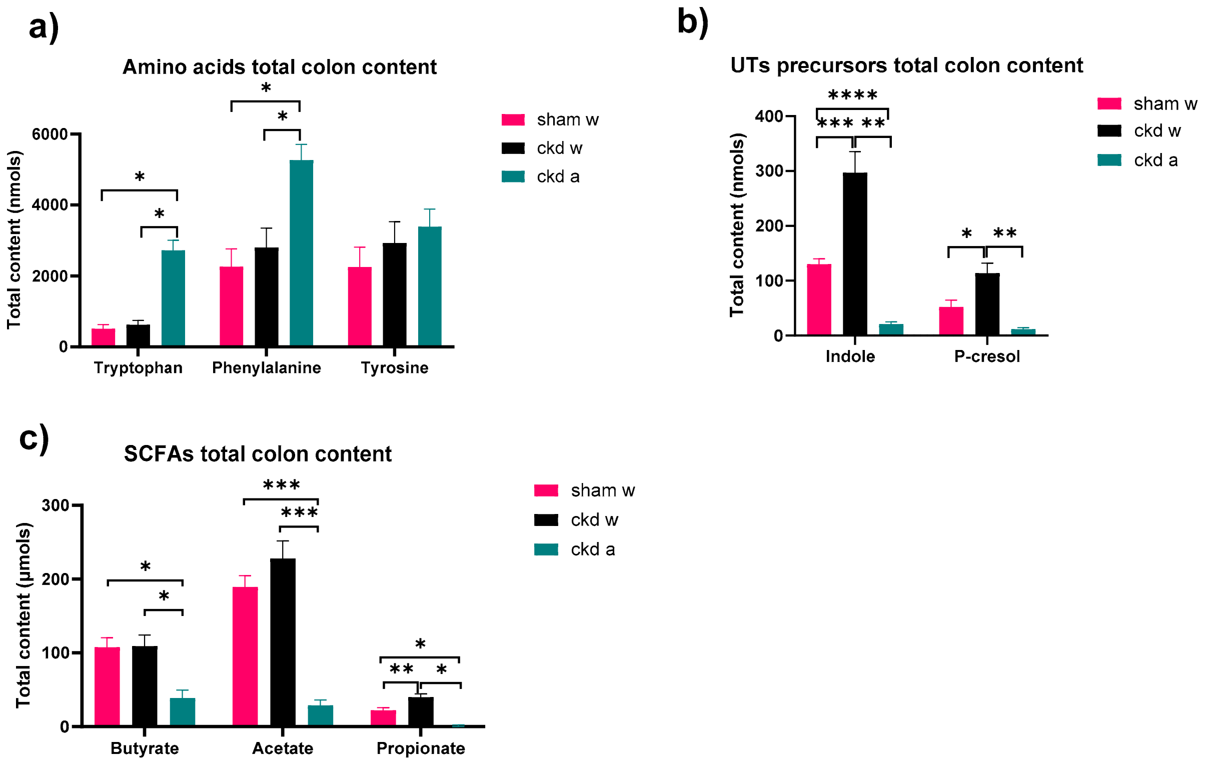

Figure S5. Colon concentration of a) amino acids; b) uremic toxins (UTs) precursors; c) short-chain fatty acids (SCFAs) in each section of the colon of the rats. Mixed-effects model with Geisser-Greenhouse correction (matched values) with Tukey's multiple comparisons test was applied for comparisons between the three groups of rats and the different sections. Mean ± SEM are shown. *, # and § show the post hoc test between the three groups: * = CKD A compared to SHAM W. # = CKD A compared to CKD W. § =CKD W compared to SHAM W. * or # or § = $p<0.05$; ** or ## or §§ = $p<0.01$; *** or ### or §§§ = $p<0.001$; **** or #### or §§§§ = $p<0.0001$.

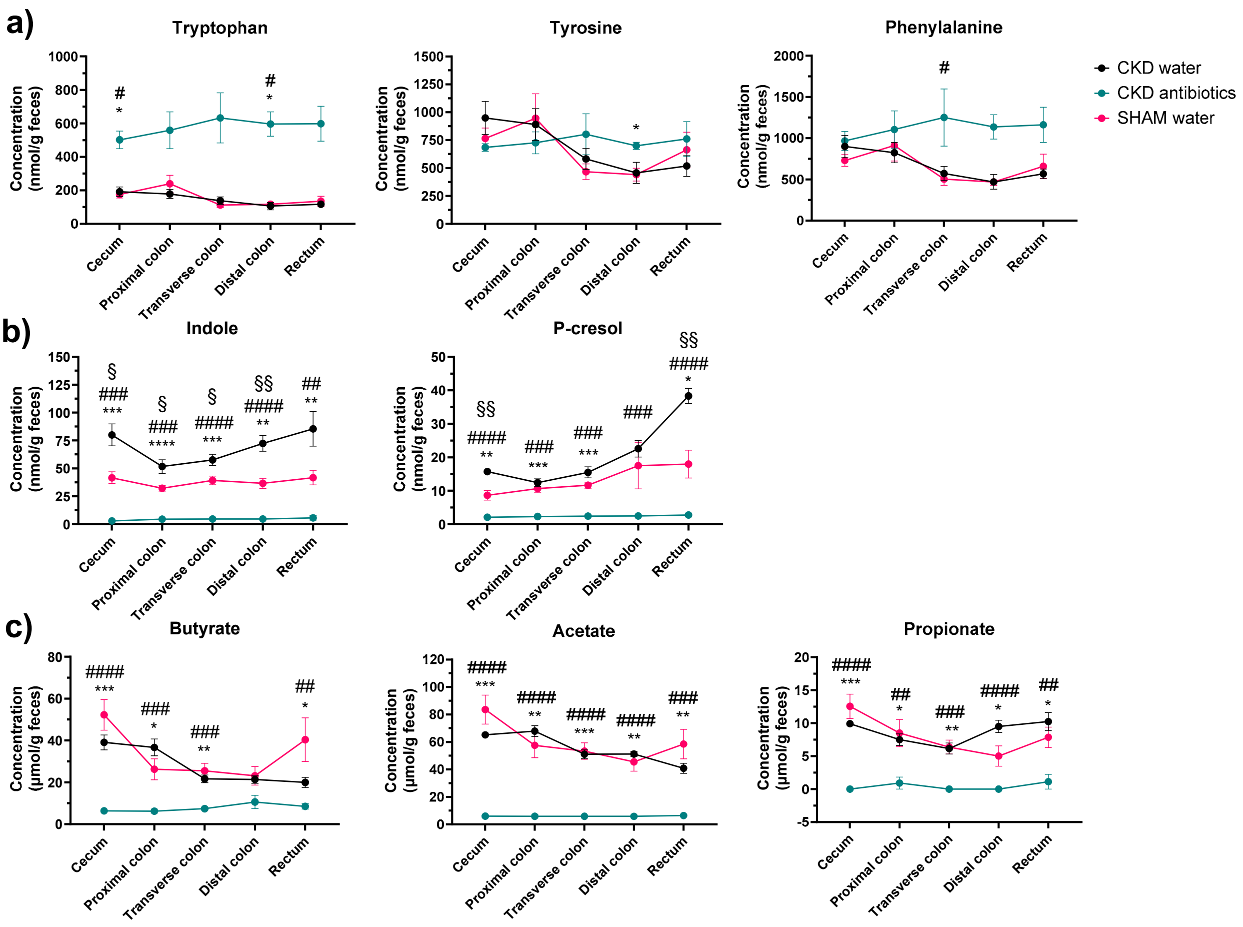

Figure S6. A) 24-hours urinary amino acids in control animals, CKD animals, and CKD animals treated with antibiotics. B) 24-hours urinary amino acid-derived metabolites in rats. Mann-Whitney or Unpaired Student t test was used for pairwise comparisons. Median ± interquartile range are shown for non-parametric data. Mean ± SEM are shown for parametric data. *p<0.01; **p<0.005

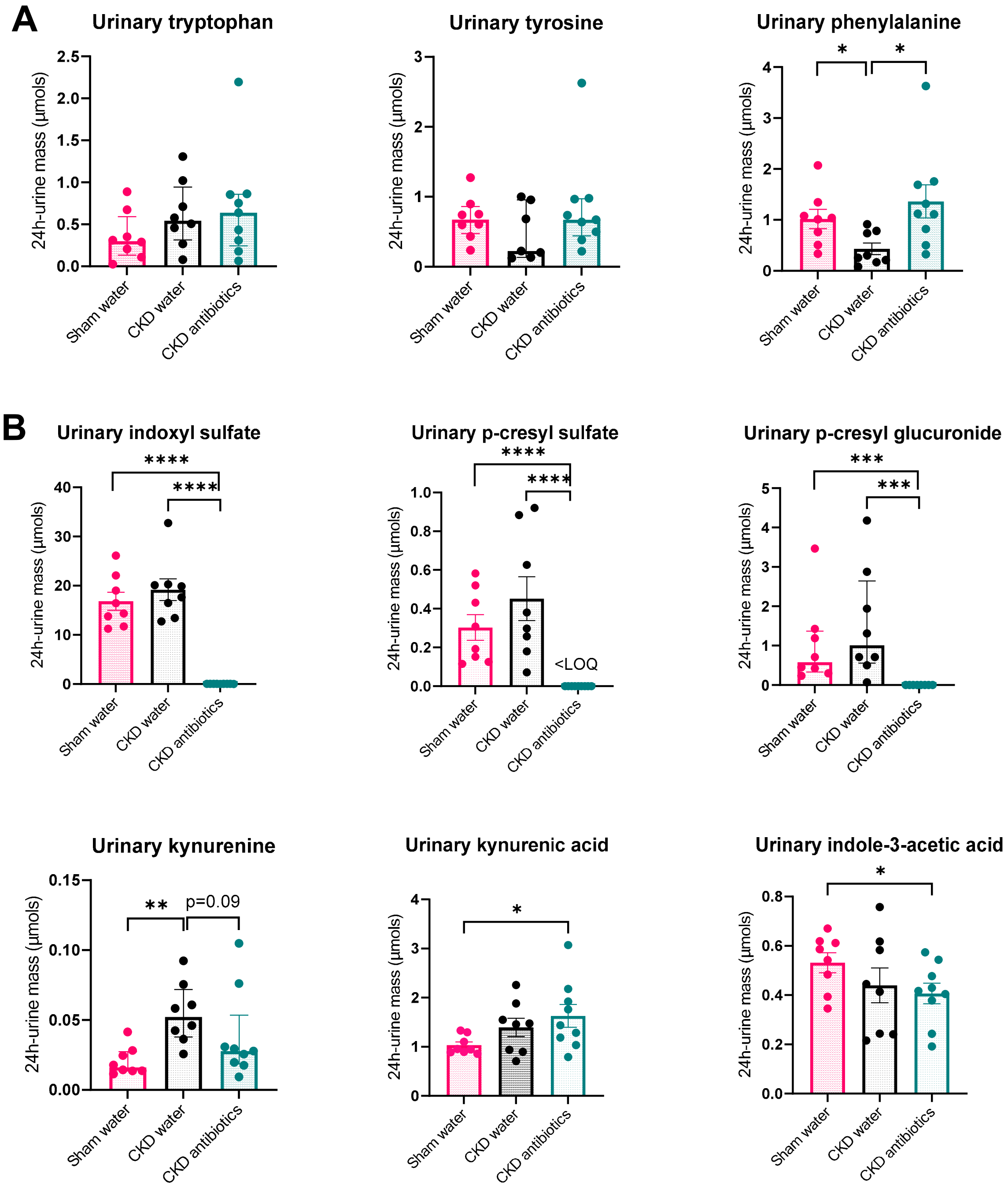

SUPPLEMENTARYTABLES

Table S1. LOD and LOQ of uremic toxins measured with LC-MS/MS.

	μM		
	**Range**	**LOQ**	**LOD**
Tryptophan	184-0.09	0,14	0,05
Phenylalanine	325-0.16	0,16	0,05
Tyrosine	186-0.09	0,6	0,05
P-cresyl glucuronide	72-0.035	0,035	0,01
Indoxyl sulfate	349-0.17	0,17	0,03
P-cresyl sulfate	597-0.29	0,29	0,03
Kynurenine	20.6-0.01	0,01	0,01
Kynurenic Acid	12.4-0.006	0,012	0,006
Indole-3-acetic acid	62-0.03	0,062	0,02

Table S2. Summary of rat samples/specimen analyzed

	**Analysis**
**Plasma**	Creatinine, urea, uremic toxins (indoxyl sulfate, p-cresyl sulfate, p-cresyl glucuronide, kynurenine, kynurenic acid, indole-3-acetic acid, amino acids (tryptophan, tyrosine, phenylalanine)
**Urine**	Creatinine, urea, uremic toxins (indoxyl sulfate, p-cresyl sulfate, p-cresyl glucuronide, kynurenine, kynurenic acid, indole-3-acetic acid, amino acids (tryptophan, tyrosine, phenylalanine)
**Colon content and fresh feces**	SCFAs (butyrate, acetate, propionate) and uremic toxin precursors (indole and p-cresol)
